# 5-Chloro-2-phenyl-1,3-benzothia­zole

**DOI:** 10.1107/S1600536812036057

**Published:** 2012-08-31

**Authors:** Sammer Yousuf, Shazia Shah, Nida Ambreen, Khalid M. Khan, Shakil Ahmed

**Affiliations:** a H. E. J. Research Institute of Chemistry, International Center for Chemical and Biological Sciences, University of Karachi, Karachi 75270, Pakistan

## Abstract

In the structure of the title compound, C_13_H_8_ClNS, the dihedral angle between the benzothia­zole ring system and the phenyl ring is 7.11 (8)°. In the crystal, mol­ecules are arranged parallel to the *c* axis.

## Related literature

For biological activites of benzothia­zole compounds, see: Venkatesh & Pandeya (2009 ▶); Sreenivasa *et al.* (2009 ▶); Kok *et al.* (2008 ▶); Siddiqui *et al.* (2007 ▶); Maharan *et al.* (2007 ▶); Pattan *et al.* (2005 ▶); Hout *et al.* (2004 ▶); Chohan *et al.* (2003 ▶); Bénéteau *et al.* (1999 ▶). For the crystal structure of benzothia­zole derivatives, see: Lakshmanan *et al.* (2011 ▶); Zhang *et al.* (2008 ▶).
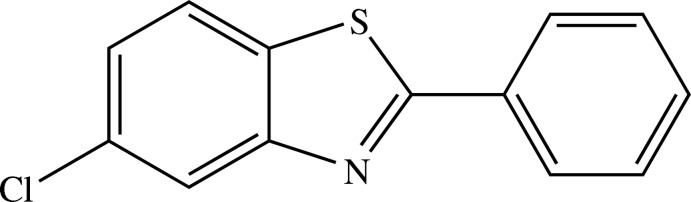


## Experimental

### 

#### Crystal data

C_13_H_8_ClNS
*M* *_r_* = 245.71
Monoclinic, 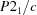

*a* = 7.4057 (9) Å
*b* = 5.9100 (7) Å
*c* = 25.165 (3) Å
β = 93.402 (3)°
*V* = 1099.5 (2) Å^3^
*Z* = 4
Mo *K*α radiation
μ = 0.50 mm^−1^
*T* = 273 K
0.36 × 0.13 × 0.09 mm

#### Data collection

Bruker SMART APEX CCD area-detector diffractometer
Absorption correction: multi-scan (*SADABS*; Bruker, 2000 ▶) *T*_min_ = 0.840, *T*_max_ = 0.956
6221 measured reflections
2013 independent reflections
1706 reflections with *I* > 2σ(*I*)
*R*_int_ = 0.023

#### Refinement

*R*[*F* ^2^ > 2σ(*F* ^2^)] = 0.035
*wR*(*F* ^2^) = 0.093
*S* = 1.04
2013 reflections
145 parameters
H-atom parameters constrained
Δρ_max_ = 0.24 e Å^−3^
Δρ_min_ = −0.21 e Å^−3^

### 

Data collection: *SMART* (Bruker, 2000 ▶); cell refinement: *SAINT* (Bruker, 2000 ▶); data reduction: *SAINT*; program(s) used to solve structure: *SHELXS97* (Sheldrick, 2008 ▶); program(s) used to refine structure: *SHELXL97* (Sheldrick, 2008 ▶); molecular graphics: *SHELXTL* (Sheldrick, 2008 ▶); software used to prepare material for publication: *SHELXTL*, *PARST* (Nardelli, 1995 ▶) and *PLATON* (Spek, 2009 ▶).

## Supplementary Material

Crystal structure: contains datablock(s) global, I. DOI: 10.1107/S1600536812036057/wn2488sup1.cif

Structure factors: contains datablock(s) I. DOI: 10.1107/S1600536812036057/wn2488Isup2.hkl

Supplementary material file. DOI: 10.1107/S1600536812036057/wn2488Isup3.cml

Additional supplementary materials:  crystallographic information; 3D view; checkCIF report

## Figures and Tables

**Fig. 1.**
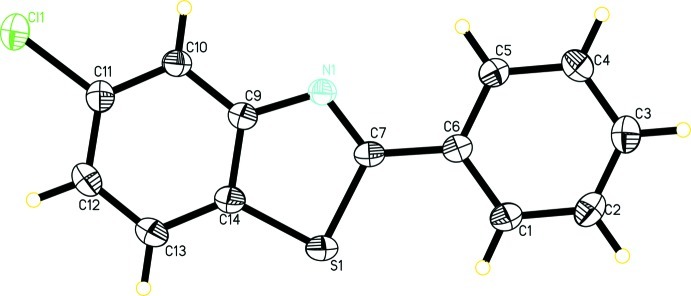
The molecular structure of the title compound with displacement ellipsoids drawn at the 30% probability level. Hydrogen atoms are shown as spheres of arbitrary radius.

**Fig. 2.**
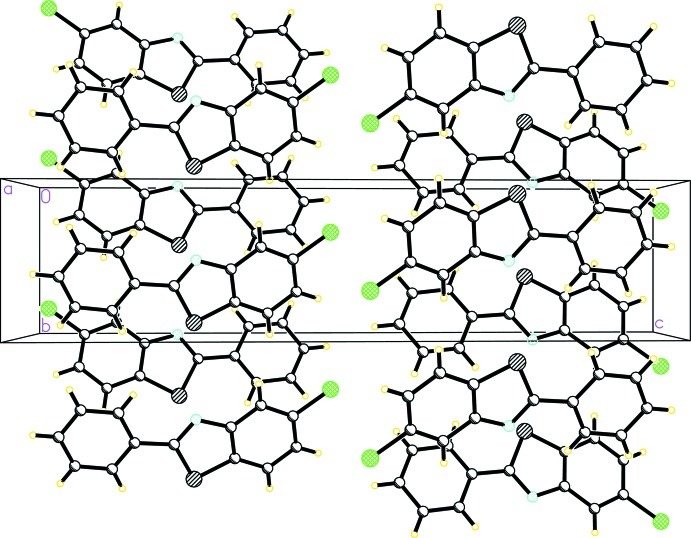
The crystal packing of the title compound.

## supplementary crystallographic information

### Comment

Benzothiazoles represent an important class of heterocyclic compounds and are known to have numerous biological activities, including antimicrobial, antimalarial, anticancer, anti-inflamatory, antidiabetic, anticonvulsant, antitumor and anthelmintic properties (Venkatesh & Pandeya, 2009; Sreenivasa *et al.*, 2009; Kok *et al.*, 2008; Siddiqui *et al.*, 2007; Maharan *et al.*, 2007; Pattan *et al.*, 2005; Hout *et al.*, 2004; Chohan *et al.*, 2003; Bénéteau *et al.*, 1999). The title compound was prepared as part of an ongoing research effort to synthesize libraries of hetereocyclic compounds and evaluate thei different biological activities.

In the structure (Fig. 1) of the title compound, C_13_H_8_ClNS, the dihedral angle between the benzothiazole ring system and the phenyl ring is 7.11 (8)°. The bond lengths and angles are similar to those in structurally related benzothiazole compounds (Lakshmanan *et al.*, 2011; Zhang *et al.*, 2008). In the crystal structure the molecules are arranged parallel to the *c*-axis (Fig. 2).

### Experimental

In a 50 ml round-bottomed flask 2-amino-4-chlorobenzenethiol (0.159 g, 1 mmol), benzaldehyde (0.106 g, 1 mmol), *N*,*N*-dimethylformamide (10 ml), and sodium metabisulfite (0.2 g) were added with continuous stirring and allowed to reflux for 2 h. Progress of the reaction was monitored by TLC. After completion of the reaction, the mixture was allowed to cool at room temperature and addition of cold water produced a solid precipitate. Crystallization from ethanol afforded pure crystals of the title compound (0.245 g, 91.8% yield); these were found to be suitable for single-crystal X-ray diffraction studies.

### Refinement

H atoms were positioned geometrically and constrained to ride on their parent atoms, with Csp^2^—H = 0.93 Å and *U*_iso_(H)= 1.2*U*_eq_(C).

### Crystal data

**Table:**

C_13_H_8_ClNS	*F*(000) = 504
*M**_r_* = 245.71	*D*_x_ = 1.484 Mg m^−^^3^
Monoclinic, *P*2_1_/*c*	Mo *K*α radiation, λ = 0.71073 Å
*a* = 7.4057 (9) Å	Cell parameters from 2167 reflections
*b* = 5.9100 (7) Å	θ = 3.1–28.2°
*c* = 25.165 (3) Å	µ = 0.50 mm^−^^1^
β = 93.402 (3)°	*T* = 273 K
*V* = 1099.5 (2) Å^3^	Plate, colorless
*Z* = 4	0.36 × 0.13 × 0.09 mm

### Data collection

**Table:**

Bruker SMART APEX CCD area-detector diffractometer	2013 independent reflections
Radiation source: fine-focus sealed tube	1706 reflections with *I* > 2σ(*I*)
Graphite monochromator	*R*_int_ = 0.023
ω scan	θ_max_ = 25.5°, θ_min_ = 2.8°
Absorption correction: multi-scan (*SADABS*; Bruker, 2000)	*h* = −8→8
*T*_min_ = 0.840, *T*_max_ = 0.956	*k* = −7→6
6221 measured reflections	*l* = −30→30

### Refinement

**Table:**

Refinement on *F*^2^	Primary atom site location: structure-invariant direct methods
Least-squares matrix: full	Secondary atom site location: difference Fourier map
*R*[*F*^2^ > 2σ(*F*^2^)] = 0.035	Hydrogen site location: inferred from neighbouring sites
*wR*(*F*^2^) = 0.093	H-atom parameters constrained
*S* = 1.04	*w* = 1/[σ^2^(*F*_o_^2^) + (0.0522*P*)^2^ + 0.1614*P*] where *P* = (*F*_o_^2^ + 2*F*_c_^2^)/3
2013 reflections	(Δ/σ)_max_ < 0.001
145 parameters	Δρ_max_ = 0.24 e Å^−^^3^
0 restraints	Δρ_min_ = −0.21 e Å^−^^3^

### Special details

**Table:**

Geometry. All e.s.d.'s (except the e.s.d. in the dihedral angle between two l.s. planes) are estimated using the full covariance matrix. The cell e.s.d.'s are taken into account individually in the estimation of e.s.d.'s in distances, angles and torsion angles; correlations between e.s.d.'s in cell parameters are only used when they are defined by crystal symmetry. An approximate (isotropic) treatment of cell e.s.d.'s is used for estimating e.s.d.'s involving l.s. planes.
Refinement. Refinement of *F*^2^ against ALL reflections. The weighted *R*-factor *wR* and goodness of fit *S* are based on *F*^2^, conventional *R*-factors *R* are based on *F*, with *F* set to zero for negative *F*^2^. The threshold expression of *F*^2^ > σ(*F*^2^) is used only for calculating *R*-factors(gt) *etc*. and is not relevant to the choice of reflections for refinement. *R*-factors based on *F*^2^ are statistically about twice as large as those based on *F*, and *R*- factors based on ALL data will be even larger.

### Fractional atomic coordinates and isotropic or equivalent isotropic displacement parameters (Å^2^)

**Table:**

	*x*	*y*	*z*	*U*_iso_*/*U*_eq_	
Cl1	0.26215 (9)	−0.18042 (11)	0.02881 (2)	0.0660 (2)	
S1	0.33963 (7)	0.39652 (8)	0.234075 (19)	0.04489 (18)	
N1	0.19011 (19)	−0.0014 (3)	0.22680 (5)	0.0367 (4)	
C1	0.2624 (3)	0.3164 (3)	0.35279 (8)	0.0449 (5)	
H1B	0.3131	0.4479	0.3399	0.054*	
C2	0.2426 (3)	0.2922 (4)	0.40663 (8)	0.0519 (5)	
H2A	0.2800	0.4079	0.4298	0.062*	
C3	0.1683 (3)	0.0995 (4)	0.42641 (8)	0.0514 (5)	
H3A	0.1555	0.0846	0.4628	0.062*	
C4	0.1123 (3)	−0.0732 (4)	0.39196 (8)	0.0484 (5)	
H4A	0.0617	−0.2040	0.4052	0.058*	
C5	0.1315 (2)	−0.0515 (3)	0.33809 (7)	0.0419 (4)	
H5A	0.0941	−0.1681	0.3152	0.050*	
C6	0.2067 (2)	0.1442 (3)	0.31764 (7)	0.0361 (4)	
C7	0.2333 (2)	0.1595 (3)	0.26047 (7)	0.0349 (4)	
C9	0.2440 (2)	0.0541 (3)	0.17678 (7)	0.0353 (4)	
C10	0.2208 (2)	−0.0858 (3)	0.13211 (7)	0.0395 (4)	
H10A	0.1639	−0.2256	0.1340	0.047*	
C11	0.2850 (3)	−0.0087 (3)	0.08523 (7)	0.0435 (5)	
C12	0.3695 (3)	0.2000 (4)	0.08090 (8)	0.0477 (5)	
H12A	0.4101	0.2459	0.0483	0.057*	
C13	0.3932 (3)	0.3391 (3)	0.12465 (8)	0.0457 (5)	
H13A	0.4498	0.4789	0.1222	0.055*	
C14	0.3300 (2)	0.2643 (3)	0.17274 (7)	0.0383 (4)	

### Atomic displacement parameters (Å^2^)

**Table:**

	*U*^11^	*U*^22^	*U*^33^	*U*^12^	*U*^13^	*U*^23^
Cl1	0.0921 (5)	0.0674 (4)	0.0395 (3)	−0.0125 (3)	0.0133 (3)	−0.0074 (2)
S1	0.0502 (3)	0.0336 (3)	0.0509 (3)	−0.0091 (2)	0.0039 (2)	−0.0026 (2)
N1	0.0381 (9)	0.0326 (9)	0.0397 (8)	−0.0015 (6)	0.0048 (6)	−0.0006 (6)
C1	0.0482 (12)	0.0366 (11)	0.0496 (11)	−0.0008 (9)	0.0011 (8)	−0.0052 (8)
C2	0.0564 (13)	0.0527 (14)	0.0458 (11)	0.0039 (10)	−0.0028 (9)	−0.0121 (9)
C3	0.0522 (12)	0.0608 (14)	0.0411 (10)	0.0081 (10)	0.0009 (9)	0.0016 (9)
C4	0.0495 (13)	0.0461 (12)	0.0497 (11)	0.0014 (9)	0.0033 (9)	0.0089 (9)
C5	0.0422 (11)	0.0392 (11)	0.0440 (10)	−0.0006 (8)	−0.0007 (8)	−0.0034 (8)
C6	0.0307 (10)	0.0349 (10)	0.0422 (10)	0.0030 (7)	−0.0006 (7)	−0.0025 (7)
C7	0.0282 (9)	0.0308 (10)	0.0453 (10)	0.0003 (7)	−0.0003 (7)	−0.0011 (7)
C9	0.0305 (9)	0.0334 (10)	0.0423 (9)	0.0018 (7)	0.0032 (7)	0.0024 (7)
C10	0.0418 (11)	0.0343 (10)	0.0427 (10)	−0.0029 (8)	0.0041 (8)	0.0000 (8)
C11	0.0456 (12)	0.0464 (12)	0.0388 (10)	0.0020 (9)	0.0045 (8)	0.0000 (8)
C12	0.0472 (12)	0.0511 (13)	0.0457 (11)	0.0002 (9)	0.0102 (8)	0.0110 (9)
C13	0.0435 (11)	0.0398 (11)	0.0545 (11)	−0.0034 (9)	0.0078 (9)	0.0080 (9)
C14	0.0339 (10)	0.0348 (10)	0.0462 (10)	0.0002 (8)	0.0025 (7)	0.0014 (8)

### Geometric parameters (Å, º)

**Table:**

Cl1—C11	1.7453 (19)	C4—H4A	0.9300
S1—C14	1.7279 (18)	C5—C6	1.395 (3)
S1—C7	1.7566 (18)	C5—H5A	0.9300
N1—C7	1.301 (2)	C6—C7	1.466 (2)
N1—C9	1.382 (2)	C9—C10	1.398 (2)
C1—C2	1.379 (3)	C9—C14	1.402 (2)
C1—C6	1.395 (3)	C10—C11	1.376 (2)
C1—H1B	0.9300	C10—H10A	0.9300
C2—C3	1.371 (3)	C11—C12	1.390 (3)
C2—H2A	0.9300	C12—C13	1.377 (3)
C3—C4	1.386 (3)	C12—H12A	0.9300
C3—H3A	0.9300	C13—C14	1.395 (3)
C4—C5	1.377 (3)	C13—H13A	0.9300
			
C14—S1—C7	88.93 (8)	N1—C7—S1	115.77 (13)
C7—N1—C9	110.29 (15)	C6—C7—S1	120.63 (13)
C2—C1—C6	120.18 (19)	N1—C9—C10	124.31 (16)
C2—C1—H1B	119.9	N1—C9—C14	115.66 (15)
C6—C1—H1B	119.9	C10—C9—C14	120.02 (15)
C3—C2—C1	120.75 (19)	C11—C10—C9	117.48 (18)
C3—C2—H2A	119.6	C11—C10—H10A	121.3
C1—C2—H2A	119.6	C9—C10—H10A	121.3
C2—C3—C4	119.73 (18)	C10—C11—C12	122.76 (17)
C2—C3—H3A	120.1	C10—C11—Cl1	118.90 (15)
C4—C3—H3A	120.1	C12—C11—Cl1	118.33 (14)
C5—C4—C3	120.16 (19)	C13—C12—C11	120.25 (17)
C5—C4—H4A	119.9	C13—C12—H12A	119.9
C3—C4—H4A	119.9	C11—C12—H12A	119.9
C4—C5—C6	120.47 (18)	C12—C13—C14	118.07 (18)
C4—C5—H5A	119.8	C12—C13—H13A	121.0
C6—C5—H5A	119.8	C14—C13—H13A	121.0
C1—C6—C5	118.72 (17)	C13—C14—C9	121.40 (17)
C1—C6—C7	121.69 (17)	C13—C14—S1	129.28 (15)
C5—C6—C7	119.52 (15)	C9—C14—S1	109.32 (12)
N1—C7—C6	123.50 (16)		
			
C6—C1—C2—C3	0.1 (3)	C7—N1—C9—C14	−0.3 (2)
C1—C2—C3—C4	0.0 (3)	N1—C9—C10—C11	−178.70 (17)
C2—C3—C4—C5	0.1 (3)	C14—C9—C10—C11	0.1 (3)
C3—C4—C5—C6	−0.2 (3)	C9—C10—C11—C12	−0.4 (3)
C2—C1—C6—C5	−0.1 (3)	C9—C10—C11—Cl1	178.89 (14)
C2—C1—C6—C7	−177.15 (17)	C10—C11—C12—C13	0.5 (3)
C4—C5—C6—C1	0.2 (3)	Cl1—C11—C12—C13	−178.86 (15)
C4—C5—C6—C7	177.29 (16)	C11—C12—C13—C14	−0.2 (3)
C9—N1—C7—C6	−175.21 (15)	C12—C13—C14—C9	−0.2 (3)
C9—N1—C7—S1	1.28 (19)	C12—C13—C14—S1	179.72 (15)
C1—C6—C7—N1	177.03 (17)	N1—C9—C14—C13	179.11 (17)
C5—C6—C7—N1	0.0 (3)	C10—C9—C14—C13	0.2 (3)
C1—C6—C7—S1	0.7 (2)	N1—C9—C14—S1	−0.8 (2)
C5—C6—C7—S1	−176.27 (14)	C10—C9—C14—S1	−179.71 (14)
C14—S1—C7—N1	−1.50 (15)	C7—S1—C14—C13	−178.71 (18)
C14—S1—C7—C6	175.10 (14)	C7—S1—C14—C9	1.20 (14)
C7—N1—C9—C10	178.56 (17)		

## References

[bb1] Bénéteau, V., Besson, T., Guillard, J., Léonce, S. & Pfeiffer, B. (1999). *Eur. J. Med. Chem.* **34**, 1053–1060.

[bb2] Bruker (2000). *SADABS*, *SMART* and *SAINT* Bruker AXS Inc., Madison, Wisconsin, USA.

[bb3] Chohan, Z. H., Pervez, H., Scozzafava, A. & Supuran, C. T. (2003). *J. Chem. Soc. Pak.* **25**, 308–313.

[bb4] Hout, S., Azas, N., Darque, A., Robin, M., Di Giorgio, C., Gasquet, M., Galy, J. & David, P. (2004). *Parasitology*, **129**, 525–542. 10.1017/s0031182004006031 15552398

[bb5] Kok, S. H. L., *et al.* (2008). *Bioorg. Med. Chem.* **16**, 3626–3631. 10.1016/j.bmc.2008.02.005 18295491

[bb6] Lakshmanan, D., Raj, R. M., Selvakumar, R., Bakthadoss, M. & Murugavel, S. (2011). *Acta Cryst.* E**67**, o2259. 10.1107/S160053681103114X PMC3200763 22058918

[bb7] Maharan, M. A., William, S., Ramzy, F. & Sembel, A. M. (2007). *Molecules*, **12**, 622–633. 10.3390/12030622 PMC6270298 17851416

[bb8] Nardelli, M. (1995). *J. Appl. Cryst.* **28**, 659.

[bb9] Pattan, S. R., Suresh, C., Pujar, V. D., Reddy, V. V. K., Rasal, V. P. & Koti, B. C. (2005). *Indian J. Chem. Sect. B*, **44**, 2404–2408.

[bb10] Sheldrick, G. M. (2008). *Acta Cryst.* A**64**, 112–122. 10.1107/S0108767307043930 18156677

[bb11] Siddiqui, N., Pandeya, S. N., Khan, S. A., Stables, J., Rana, A., Alam, M., Arshad, M. F. & Bhat, M. A. (2007). *Bioorg. Med. Chem. Lett.* **17**, 255–259. 10.1016/j.bmcl.2006.09.053 17046248

[bb12] Spek, A. L. (2009). *Acta Cryst.* D**65**, 148–155. 10.1107/S090744490804362X PMC2631630 19171970

[bb13] Sreenivasa, M., Jaychand, E., Shivakumar, B., Jayrajkumar, K. & Vijaykumar, J. (2009). *Arch. Pharm. Sci. Res.* **1**, 150–157.

[bb14] Venkatesh, P. & Pandeya, S. N. (2009). *Int. J. ChemTech. Res.* **1**, 1354–1358.

[bb15] Zhang, Y., Su, Z.-H., Wang, Q.-Z. & Teng, L. (2008). *Acta Cryst.* E**64**, o2065. 10.1107/S1600536808031565 PMC2959689 21580931

